# Non-invasive markers of liver fibrosis in fatty liver disease are unreliable in people of South Asian descent

**DOI:** 10.1136/flgastro-2017-100865

**Published:** 2017-11-16

**Authors:** Sampath De Silva, Wenhao Li, Polychronis Kemos, James H Brindley, Jibran Mecci, Salma Samsuddin, Joanne Chin-Aleong, Roger M Feakins, Graham R Foster, Wing-Kin Syn, William Alazawi

**Affiliations:** 1 Liver Unit, Blizard Institute, QueenMary University of London, London, UK; 2 Department of Histopathology, Bart’s Health NHS Trust, London, UK; 3 Section of Gastroenterology, Ralph H Johnson Veterans Affairs Medical Center, Charleston, USA; 4 Division of Gastroenterology and Hepatology, Medical University of South Carolina, Charleston, USA

**Keywords:** nonalcoholic steatohepatitis, fibrosis, fatty liver, liver biopsy

## Abstract

**Objective:**

Liver biopsy is the most accurate method for determining stage and grade of injury in non-alcoholic fatty liver disease (NAFLD). Given risks and limitations of biopsy, non-invasive tests such as NAFLD fibrosis score, aspartate transaminase (AST) to platelet ratio index, Fib-4, AST/alanine transaminase ratio and BARD are used. Prevalence and severity of NAFLD and metabolic syndrome vary by ethnicity, yet tests have been developed in largely white populations. We tested our hypothesis that non-invasive tests that include metabolic parameters are less accurate in South Asian compared with white patients.

**Design:**

Retrospective cross-sectional.

**Setting:**

Specialist liver centre.

**Patients:**

Patients with histologically confirmed NAFLD.

**Interventions:**

Scores calculated using clinical data taken within 1 week and compared with histology (Kleiner).

**Main outcome measures:**

Diagnostic test characteristics.

**Results:**

175 patients were identified. South Asians (n=90) were younger, had lower body mass index and lower proportion of obesity compared with white patients (n=79), with comparable rates of diabetes and liver injury. Tests are less sensitive at detecting advanced fibrosis in South Asian compared with white patients. Relative risk of correct diagnosis in white patients compared with South Asians is 1.86 (95% CI 1.4 to 2.6). In binary logistic regression models, ethnicity and platelet count predicted accuracy. Transient elastography was equally and highly accurate in both ethnicities.

**Conclusions:**

Blood test-based non-invasive scores are less accurate in South Asian patients, irrespective of metabolic parameters. Ethnicity should be considered when devising risk-stratification algorithms for NAFLD.

## Introduction

Non-alcoholic fatty liver disease (NAFLD) affects up to 30% of the general population^1^ and is the hepatic manifestation of the metabolic syndrome. NAFLD is a spectrum of diseases that encompasses simple steatosis, non-alcoholic steatohepatitis (NASH) and fibrosis, which can lead to cirrhosis, liver failure and hepatocellular carcinoma.^2^ However, not all patients progress through the full hepatological spectrum of NAFLD.^3 4^ Determinants of progression include diabetes, diet and ethnicity but the most accurate predictor of liver-related mortality is presence of liver fibrosis on biopsy.^5–7^

It is neither feasible nor desirable to perform a liver biopsy in every patient with suspected NAFLD because the procedure is invasive, associated with potential complications, cost, sampling error and interobserver variability. Thus, non-invasive liver tests (NILTs) have been developed as an alternative to liver biopsy. These can be biomarker based^8–10^ or based on routinely collected clinical and laboratory data such as NAFLD fibrosis score (NFS),^11^ Fib-4,^12^ BARD,^13^ aspartate transaminase (AST) to platelet ratio index (APRI) and the AST/alanine transaminase (ALT) ratio.^14^ Liver stiffness, measured by transient elastography (TE),^15^ acoustic radiation force impulse^16^ or MRI,^17^ can be a surrogate marker of fibrosis, but requires specialist equipment and/or skilled personnel to conduct the tests.

Current guidance advocates use of these NILTs to stratify patients into specialist hepatology versus non-specialist or primary care.^18^ However, the applicability of blood test-based NILTs to different patient groups—including those of different ethnicities—has yet to be determined. Most of the work done to develop and validate these scores has been in largely white Caucasian populations.^11^ Our group and others find that the prevalence of NAFLD varies by ethnic group with increased risk in patients of South Asian and Hispanic ethnicities.^19–22^ Diabetes is more common in South Asian patients and complications of obesity are evident at lower body mass indices. Diabetes and body mass index (BMI) contribute to the calculation of commonly used risk scores (NFS and BARD). Therefore, we hypothesised that these scores would be less accurate in patients of South Asian ethnicity compared with white patients, whereas there would be no difference in accuracy in tests that do not depend on these clinical factors: Fib-4, APRI, AST/ALT ratio and liver stiffness by TE. Surprisingly, we found reduced accuracy in all blood test-based scores but not TE in South Asian compared with white patients.

## Patients and methods

We conducted a retrospective cross-sectional study of all adult patients with a histological diagnosis of any stage of NAFLD made in our centre between 2010 and 2016. The study was approved by the Barts Health National Health Service Trust Clinical Standards and Audit Department as a service evaluation of NILTs and therefore individual informed consent was not required or taken. Patients were excluded if they had any coexisting chronic liver disease, consumed more than 21 units (168 g) of alcohol per week for men and 14 units (112 g) of alcohol per week for women. Patients with inadequate biopsy specimens (as determined by the reporting pathologist), normal histology, alternative histological diagnoses or incomplete clinical data were excluded.

Liver biopsies reported by a single histopathologist (in routine clinical care) were summarised according to the National Institutes of Health NASH clinical research network (Kleiner) criteria.^23^ Clinical and laboratory data obtained within 1 week of the biopsy included sex, age, BMI (weight (kg)/height (m^2^), alcohol consumption and diabetes status. Transient elastography was performed according to standard clinical practice. Data were obtained from clinical records and included if a valid result (successful reading rate >60% and IQR of all readings <30% of the median). Patients were classified as underweight, normal weight, overweight, obese or morbidly obese using BMI adjusted for ethnicity.^24^ We recorded self-reported ethnicity and collapsed results into four categories; South Asian (Indian, Pakistani, Bangladeshi, Sri Lankan and Nepalese), White, Black, East Asian and Other.

The NFS was calculated according to the formula: −1.675+0.037×age(years)+0.094×BMI(kg/m^2^)+1.13×impaired fasting glycaemia or diabetes (yes=1, no=0)+0.99×AST/ALT ratio−0.013×platelet (×10^9^/L)−0.66×albumin (g/dL). APRI was calculated as {(AST (IU/l)/upper limit of normal)/platelet count (x10^9^)}×100. The BARD score was calculated as the sum of three components (BMI >28=1 point, AST/ALT ratio >0.8=2 points, diabetes=1 point). The BARD score was used as intended and so the BMI threshold was not adjusted for ethnicity. The Fib-4 score was calculated as: age×AST (IU/l)/platelet count (×10^9^/L).

The overall performance of NILTs was based on comparisons between the area under the receiver–operator curves (AUROC) for patients of South Asian and white ethnicities. Relative risk for sensitivity were calculated while controlling for choice of NILT. Cochran-Mantel-Haenszel statistics were used to calculate the common relative risk while the Breslow-Day statistic test was used to assess homogeneity. Analyses were performed in SAS (Cary, North Carolina, USA), Mintab (State College, Pennsylvania, USA) and SPSS (Armonk, New York, USA), and for all tests, the significance level was α=0.05.

## Results

### NASH is more aggressive in South Asian compared with white patients

We identified 239 patients with NAFLD and no other liver diagnosis and, after excluding 64 because of inadequate specimens (n=4) or incomplete data sets (n=60), our cohort comprised 175 patients with biopsy-proven NAFLD and complete clinical data (table 1). The majority of patients were male (n=116, 66%) and most were obese (n=111, 63%). Forty-six patients (26%) had advanced fibrosis (stages 3 and 4), and 63 patients (36%) had no fibrosis, of whom 26 (15% of total) had steatosis alone.

**Table 1:** Demographic and clinicopathological characteristics of patients

	South Asian (n=90)		White (n=79)		
	Mean/Median	Range	Mean/Median	Range	p Value
Age (years)	44	24–78	52	23–74	1.2×10^–5^
Sex (%male)	73%		61%		0.08
Diabetes (%)	42%		37%		0.52
BMI (kg/m^2^)	28.6	19–42	32.8	23–52	4.0×10^–8^
Liver histology					
Total NAS	3	1–6	3	1–6	0.47
Steatosis	1	1–3	1	1–3	0.89
Lobular inflammation	1	0–2	1	0–2	0.01
Hepatocyte ballooning	1	0–2	1	0–2	0.52
Fibrosis (Kleiner)	1	0–4	1	0–4	0.60
Fibrosis stage (n) F0/F1/F2/F3/F4	34/23/11/15/17		28/23/5/12/11		
Blood results					
AST (U/l)	47	10–140	48	16–133	0.75
ALT (U/l)	76	13–262	69	12–288	0.37
Platelets x10^9^/mL	237	62–449	219	40–532	0.11
Albumin (g/L)	46	28–53	45	33–52	0.04
Non-invasive liver tests					
NAFLD fibrosis score	−2.34	−5.8–3.4	−1.31	−6.1–3.0	4.0×10^–4^
APRI	0.56	0.1–2.7	0.65	0.2–3.3	0.07
AST/ALT ratio	0.70	0.3–2.0	0.77	0.3–1.7	0.13
BARD	1	0–4	1	0–4	0.09
Fib-4	1.2	0.3–9.6	1.81	0.5–10.5	0.01

Characteristics are summarised as mean for continuous data (age, BMI, blood results, NAFLD fibrosis score, APRI, AST/ALT and Fib-4) and as median for categorical data (histology and BARD score). Two-tailed p values compare South Asian and white patients using Student’s t-test for continuous and the Mann-Whitney test for categorical data. Proportions of patients who are male and who have diabetes are compared using the χ^2^ test.

ALT, alanine aminotransferase; APRI, aspartate transaminase (AST) to platelet ratio index; AST, aspartate aminotransferase; BMI, body mass index; NAFLD, non-alcoholic fatty liver disease; NAS, NAFLD activity score.

The two largest ethnic groups in our cohort were South Asian (n=90) and white (n=79) patients (table 1). The remainder were of East Asian (n=3), black (n=2) or other (n=1) ethnicities. Although there were no significant differences in the median grade or stage of liver injury, South Asians were almost a decade younger than white patients (43.6 vs 51.8 years, p=1.2×10^-5^). The mean BMI of South Asian patients (28.6 kg/m^2^) was lower than that of white patients (32.8 kg/m^2^, p=4.0×10^-8^). Using ethnicity-adjusted thresholds,^24^ proportionally fewer South Asian patients were obese (60% with BMI >27.5 kg/m^2^) compared with white patients (75% with BMI >30 kg/m^2^, p<0.05).

Given this, it was not surprising that the mean NFS (which is calculated using both age and BMI) was significantly lower in South Asian patients (table 1, p=4.0×10^-4^). The same was true for Fib-4 (p=0.01) but this did not reach statistical significance for AST/ALT, APRI or BARD. There was a positive association for each NILT with fibrosis score (see online supplementary figure 1) but the correlation was consistently higher in white patients than South Asians (see online supplementary table 1).

10.1136/flgastro-2017-100865.supp1 Supplementary file 1

### NILTs are less sensitive at detecting advanced fibrosis in South Asian patients

**Figure 1:**
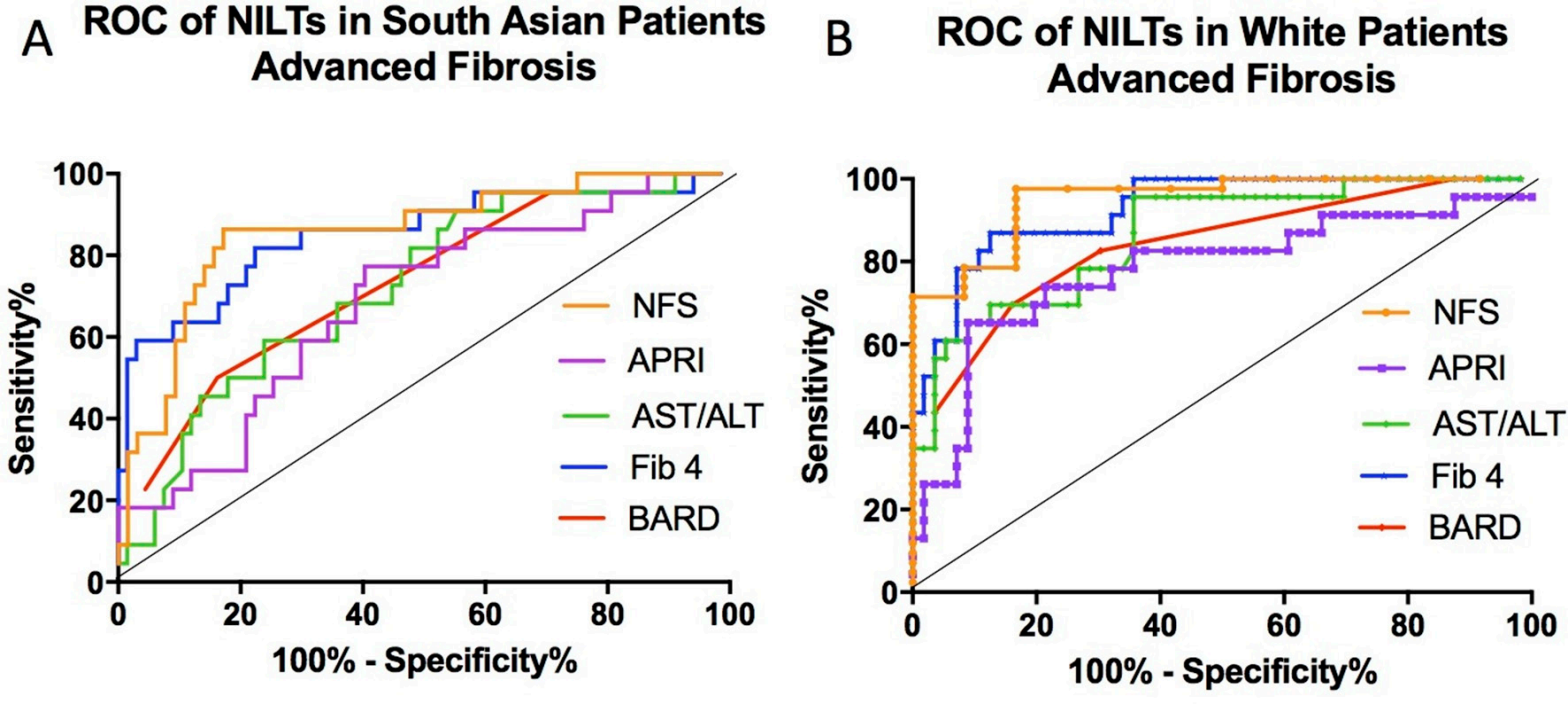
Receiver operating characteristic (ROC) curves for non-invasive liver tests. The diagnostic characteristics are shown for all five tests when used to assess advanced fibrosis (Kleiner stage 3 or 4) in patients of South Asian (A) or white (B) ethnicities. NFS, non-alcoholic fatty liver disease fibrosis score; APRI, aspartate aminotransferase to platelet ratio index, AST/ALT, aspartate aminotransferase to alanine aminotransferase ratio.

The AUROC was calculated for each test’s ability to identify patients with advanced fibrosis (figure 1) using standard cut-off values (NFS >0.676, APRI >1.0, AST/ALT ratio >0.8, BARD >2 and Fib-4 >3.25). In white patients, the NFS was most accurate at predicting advanced fibrosis (AUROC 0.95), followed by Fib-4, BARD, APRI and AST/ALT ratio (see online supplementary table 2). The AUROC for all tests was lower in South Asian compared with white patients (Friedman: p=0.025) but the two most accurate scores in South Asians were still the NFS and Fib-4. Taken together, all five NILTs and not only those that contain components of the metabolic syndrome are less accurate in patients of South Asian compared with white ethnicity. NILTs were less sensitive in South Asian (range 0.09–0.52) compared with white patients (range 0.50–0.75), but specificities were comparable.

Having confirmed homogeneity of relative risks, (p=0.61; Breslow-Day test), we found a strong association between ethnicity and NILT sensitivity (p<0.0001; Cochran-Mantel-Haenszel test). The relative risk of a true positive finding of advanced fibrosis is 1.85 (95% CI 1.35 to 2.56) in white compared with South Asian patients (see online supplementary figure 2).

### Accuracy of NILTs is affected by ethnicity, platelet count and albumin

To identify the factors that independently determine the accuracy of each NILT in our population, we performed analyses of association between a range of clinical and pathological parameters including ethnicity with correct diagnosis of fibrosis (see online supplementary table 3). Albumin and ALT or AST were significantly associated with correct diagnosis for all five NILTs. Other variables of note included platelet count, BMI and ethnicity. Ethnicity had a significant impact on accuracy of NFS and APRI, but the association with Fib-4 did not reach statistical significance (p=0.065).

**Figure 2:**
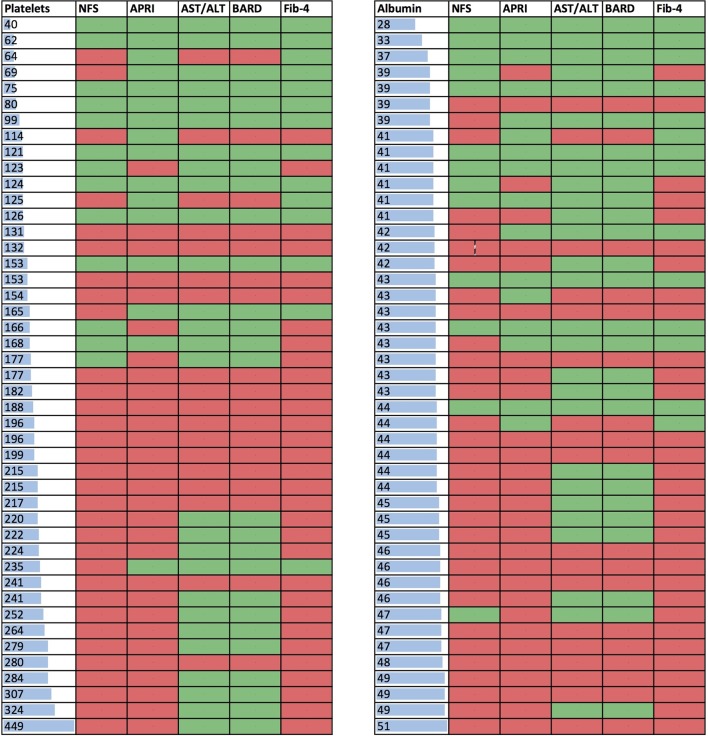
Heat maps showing the effect of platelets (x10**^9^/**L; left) and albumin (g/L; right) on NILT accuracy in 46 patients of all ethnic groups with advanced fibrosis (Kleiner stage 3 or 4). Each row represents a single patient and shows the platelet count (left-hand heat map) or serum albumin concentration (right-hand heat map) is shown for that patient. Each column represents one of the five NILTs. A green cell in a column indicates that the test has correctly classified the patient as having advanced fibrosis and a red cell that the test has incorrectly classified the patient as not having advanced fibrosis. NFS, non-alcoholic fatty liver disease fibrosis score; APRI, aspartate aminotransferase to platelet ratio index, AST/ALT, aspartate aminotransferase to alanine aminotransferase ratio.

All variables that were significantly associated with NILT accuracy were considered for binary logistic regression models. The factors that predict the likelihood of the three most commonly used NILTs (NFS, APRI and Fib-4) in accurately detecting advanced fibrosis were platelet count, ethnicity and serum ALT (see online supplementary table 4). The impact of ethnicity was greatest on the NFS with borderline significance for an independent effect on APRI and Fib-4. There is a clear association of NILT accuracy with lower platelet counts and albumin levels (figure 2) although we did not include both in the regression models due to the strong correlation between both platelets and albumin levels with advanced fibrosis (and each other). NILTs (especially NFS, Fib-4 and APRI) are less likely to be accurate if the platelet count is over 150×10^9^/L with 30% of tests correctly identifying advanced fibrosis compared with 69% in patients with platelet count below 150×10^9^/L (p<0.0001). Similarly, NILTs are less likely to be accurate if the albumin is ≥43 g/L with 30% of tests correctly identifying advanced fibrosis compared with 68% in patients with albumin below 43 g/L (p<0.0001).

### Transient elastography is more accurate than blood-based NILTs in South Asian patients

**Figure 3:**
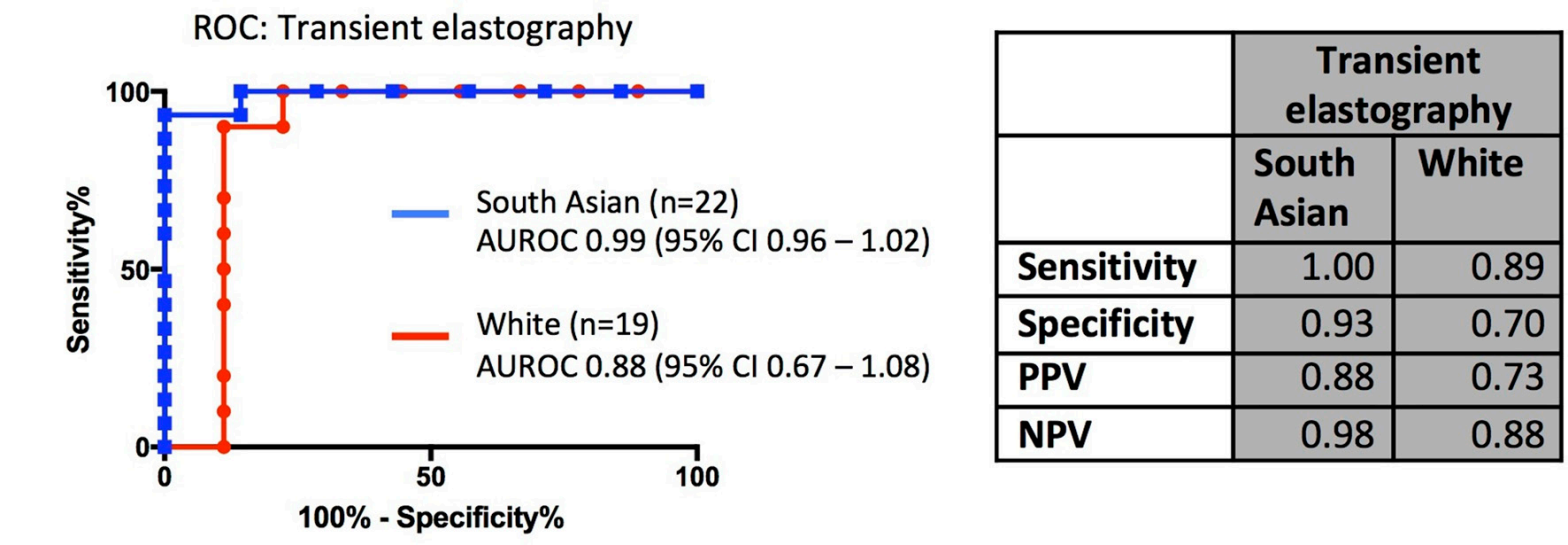
Receiver operating characteristic (ROC) curves for transient elastography. Table shows test characteristics in white and South Asian patients. AUROC, area under the receiver-operator curves; NPV, negative predictive value; PPV, positive predictive value.

Data on TE (using Fibroscan) were available for a subset of our patients (n=41). In South Asians, the AUROC for TE was much higher than other NILTs including NFS and Fib-4 (p=0.005). Using a liver stiffness of 7.9 kPa to indicate indeterminate or high risk of advanced fibrosis,^25 26^ the sensitivity of TE for detecting advanced fibrosis was higher in both ethnic groups compared with blood test-based NILTs, while maintaining specificity above 90% (figure 3).

## Discussion

The principal function of NILTs is to identify patients with high probability of having advanced fibrosis at the time of testing. Currently, decisions such as management in primary or secondary care or consideration for clinical trials are largely based on the results of these NILTs. As with any clinical test, there is a danger that if applied or interpreted inappropriately, wrong decisions can be made and, ultimately, harm may come to a patient. Despite the relatively small sample size, our data show that the sensitivity of commonly used NILTs is lower in patients of South Asian ethnicity suggesting that large numbers of South Asian patients may be inappropriately reassured that they do not have advanced disease. We recorded self-reported ethnicity and not first or subsequent generation immigration. Nevertheless, our data suggest that NASH may be more aggressive in South Asian patients who are almost a decade younger, have lower BMI and less severe BMI category than white patients with comparable disease stage. Although this has not been reported in South Asian patients before, our data are similar to those from Hispanic cohorts.^21 22 27 28^ Unfortunately, our patient cohort did not include sufficient numbers of patients of other Asian ethnic groups to comment on the differences between South Asian and, for example, East Asian ethnicities.

The factors that we identified on univariate analysis as being significantly associated with NILT accuracy (transaminases, BMI, platelet count, albumin and the presence of diabetes) are themselves components of NILT scores and are well known to be associated with an increased risk of advanced fibrosis.^29^ NILTs were designed and validated to identify patients with advanced fibrosis and it is therefore unsurprising that a NILT is more likely to be accurate if there is a priori evidence of advanced disease. For example, the heat maps in figure 2 show that NILTs are more likely to be accurate if the platelet count is below 150×10^9^/L or albumin below 43 g/L. NILTs are able to identify patients who currently have cirrhosis or advanced fibrosis and who are at risk of developing complications such as hepatocellular carcinoma—essential tools for the primary care or general physician. However, there is no evidence to suggest that they can be used to identify patients with mild or moderate fibrosis in whom current interventions (behaviour and lifestyle change) are most likely to be effective or who are the target population for the many clinical trials ongoing in NASH.

The overwhelming majority of patients in the original studies that derived NILTs were white (NFS 90%,^11^ BARD 68%^13^ and Fib-4 74%).^30^ It is therefore unsurprising that we found comparable and high sensitivities and AUROCs in white patients with these NILTs. While some validation in populations of different ethnicities has been shown,^31^ little has been done in the South Asian population, who are at increased risk of metabolic syndrome.

In our analyses, we have used the accepted high cut-offs for NFS (>0.675) and Fib-4 (>3.25). Based on the AUROC curves in figure 1, it may be possible to improve the accuracy of these two NILTs (NFS and Fib-4) in South Asian patients. New thresholds can be determined that would maximise sensitivity while maintaining high specificity, even if not to the same degree as in white patients. These new cut-offs would need validation in an independent cohort as was done for patients aged over 65.^32^

We acknowledge that this is a single-centre study of retrospectively collected routine clinical data. As a result, not all patients had a fibroscan. The biopsies were performed and read by different operators with no review by a second pathologist. The histopathologists were not blinded to the clinical features or biochemical results when reporting these cases for routine clinical practice. We have not assessed the accuracy of biomarker-based tests such as ELF^8 9^ and Fibrotest^10^ that measure proteins involved in fibrosis and extracellular matrix turnover.

A variety of guidelines for the management of NAFLD have recently been published and the majority include the NILTs studied here.^18 33^ We have shown that NILTs may be inaccurate, for example, in patients with low pretest probability of significant disease or in patients of South Asian ethnicity; however, this is not the case for TE. We propose that ethnicity should be considered a factor in the clinical decision-making process. Further studies are required to validate and to assess the accuracy, utility and cost-effectiveness of new thresholds for NFS and Fib-4, and TE in patients with NAFLD.

**Key messages**

**What is already known on this topic**

- Many guidelines advocate the use of non-invasive tests to identify patients at high risk of advanced liver disease in non-alcoholic fatty liver disease.

**What this study adds**

- Blood test-based non-invasive liver tests, but not transient elastography, are less accurate in patients of South Asian compared with white ethnicity.

**How might it impact on clinical practice in the foreseeable future**

- Clinical guidance should take ethnicity into account when recommending assessment and treatment algorithms for non-alcoholic fatty liver disease.
