# Supplementary material for: Non-invasive markers of liver fibrosis in fatty liver disease are unreliable in people of South Asian descent
Source: Frontline Gastroenterol. 2017 Nov 16;9(2):115–21. doi: 10.1136/flgastro-2017-100865 (PMC5868450; doi:10.1136/flgastro-2017-100865)
Supplement: Supplementary file 1 [file flgastro-2017-100865supp001.docx]

**Supplementary Material (Tables 1-4, Figures 1-2)**

|  | **NFS** | **APRI** | **AST/ALT** | **BARD** | **Fib-4** |
| --- | --- | --- | --- | --- | --- |
| **South Asian** | | | | | |
| **Spearman r** | 0.46 | 0.35 | 0.24 | 0.41 | 0.49 |
| **p value** | < 0.0001 | 0.0008 | 0.03 | < 0.0001 | < 0.0001 |
| **White** | | | | | |
| **Spearman r** | 0.64 | 0.42 | 0.55 | 0.54 | 0.66 |
| **p value** | < 0.0001 | < 0.0001 | < 0.0001 | < 0.0001 | < 0.0001 |

**Supplementary Table 1 Spearman Rank correlation coefficient of Non-Invasive Liver Tests with histological fibrosis score in South Asian and Asian patients.**

|  | **NFS** | | **APRI** | | **AST/ALT** | | **BARD** | | **FIB-4** | |
| --- | --- | --- | --- | --- | --- | --- | --- | --- | --- | --- |
|  | **South Asian** | **White** | **South Asian** | **White** | **South Asian** | **White** | **South Asian** | **White** | **South Asian** | **White** |
| **Advanced Fibrosis** | | | | | | | | | | |
| AUROC | 0.86 | 0.95 | 0.68 | 0.78 | 0.72 | 0.87 | 0.73 | 0.83 | 0.85 | 0.93 |
| Std Error | 0.05 | 0.03 | 0.06 | 0.06 | 0.06 | 0.04 | 0.06 | 0.05 | 0.05 | 0.03 |
| 95% CI | 0.76 - 0.95 | 0.89 – 1.00 | 0.56 - 0.81 | 0.65 - 0.91 | 0.60 - 0.84 | 0.78 - 0.95 | 0.61 - 0.85 | 0.73 - 0.94 | 0.75 - 0.96 | 0.87 – 0.99 |
| Sensitivity | 0.09 | 0.48 | 0.18 | 0.52 | 0.50 | 0.70 | 0.50 | 0.70 | 0.18 | 0.43 |
| Specificity | 0.98 | 1.00 | 0.91 | 0.91 | 0.76 | 0.82 | 0.84 | 0.84 | 1.00 | 0.98 |
| PPV | 0.67 | 1.00 | 0.40 | 0.71 | 0.41 | 0.62 | 0.50 | 0.64 | 1.00 | 0.91 |
| NPV | 0.76 | 0.82 | 0.82 | 0.82 | 0.82 | 0.87 | 0.84 | 0.87 | 0.79 | 0.81 |

**Supplementary Table 2 Characteristics of Non-Invasive Liver Tests in South Asian and White patients.**  Tests were used to identify patients with advanced fibrosis. Area under the receiver operator curve (AUROC), standard error (Std error) and 95% confidence intervals were calculated for each non-invasive test in South Asian and White patients. AUROC was are shown when tests were used to identify patients with advanced fibrosis. PPV – positive predictive value, NPV – negative predictive value.

| Variable | NFS | APRI | AST/ALT | BARD | Fib-4 |
| --- | --- | --- | --- | --- | --- |
| Chi Square correlation (p value) | | | | | |
| Ethnicity | 0.005 | 0.018 | ns | ns | ns |
| Diabetes | ns | ns | ns | ns | ns |
| Spearman correlation (r) | | | | | |
| Albumin | -0.503^**^ | -0.565^**^ | -0.413^**^ | -0.413^**^ | -0.528^**^ |
| ALT | -0.359^*^ | ns | -0.505^**^ | -0.505^**^ | ns |
| Platelets | -0.585^**^ | -0.699^**^ | ns | ns | -0.660^**^ |
| BMI | 0.493^**^ | ns | 0.451^**^ | 0.451^**^ | ns |
| Age | 0.310^*^ | ns | ns | ns | ns |
| AST | ns | 0.415^**^ | ns | ns | 0.392^**^ |

**Supplementary Table 3 Factors associated with Non-Invasive Liver Test accuracy.** Chi square (for ethnicity) and Spearman correlation co-efficient between correct identification of a patient as having advanced fibrosis and characteristics listed.

| **Significance for predictors** | | | |
| --- | --- | --- | --- |
| **Parameter** | **NFS** | **APRI** | **Fib-4** |
| **Platelets** | 0.007 | 0.007 | 0.004 |
| **Ethnicity (White)** | 0.02 | 0.05 | 0.08 |
| **ALT** | 0.03 |  |  |

**Supplementary Table 4 Logistic Regression Model.**  Developed to identify factors that predict the ability of the commonly used NILTs (NFS – NAFLD fibrosis score, APRI and Fib-4) to correctly diagnose advanced fibrosis. The model satisfies the proportional odds model (Score Test for the Proportional Odds Assumption p= 0.31).

**Supplementary Figure 1 NILT scores (y axes) are plotted against histological fibrosis score (x axes) for patients of South Asian (blue graphs) and White (red graphs) ethnicity.**

**Supplementary Figure 2 Relative risk of a true positive result when using non-invasive liver tests in White compared to South Asian patients.**
